# Tumor suppressor miR-1 restrains epithelial-mesenchymal transition and metastasis of colorectal carcinoma via the MAPK and PI3K/AKT pathway

**DOI:** 10.1186/s12967-014-0244-8

**Published:** 2014-09-08

**Authors:** Lijun Xu, Yue Zhang, Hui Wang, Guanhua Zhang, Yanqing Ding, Liang Zhao

**Affiliations:** Department of Pathology, Nanfang Hospital, Southern Medical University, Guangzhou, China; Department of Medical Oncology, Affiliated Tumor Hospital of Guangzhou Medical University, Guangzhou, China; Department of Pathology, School of Basic Medical Sciences, Southern Medical University, Guangzhou, China

**Keywords:** Colorectal carcinoma, MicroRNA, Tumor metastasis, LIM and SH3 protein 1, Signal pathway, Proteomics, Gene therapy

## Abstract

Aberrant expression of miR-1 has been implicated in various cancers. However, the mechanisms underlying the role of miR-1 in CRC progression still have not been clarified clearly. Here, we showed the decreased expression of miR-1 in colorectal carcinoma (CRC) tissues and cell lines. Ectopic introduction of miR-1 suppressed cell proliferation and migration, whereas miR-1 inhibitor performed contrary functions in CRC cells. Stable overexpression of miR-1 was sufficient to inhibit tumor growth and homing capacity *in vivo*. Proteomic analysis revealed that miR-1 modulated the expression of key cellular molecules and involved in the MAPK and PI3K/AKT pathways by inhibiting phosphorylation of ERK and AKT. Meanwhile, miR-1 also reversed epithelial–mesenchymal transition (EMT), which played a pivotal role in the initiation of metastasis. Further studies found that miR-1 can target the 3' untranslated region (3'UTR) of LIM and SH3 protein 1 (LASP1) mRNA and suppress the expression of LASP1, identified as a CRC-associated protein. In contrast to the phenotypes induced by miR-1 restoration, LASP1-induced cell proliferation and migration partly rescued miR-1-mediated biological behaviors. Our results illustrated that miR-1 play a critical role in CRC progression, which suggests its potential role in the molecular therapy of cancer.

**Electronic supplementary material:**

The online version of this article (doi:10.1186/s12967-014-0244-8) contains supplementary material, which is available to authorized users.

## Introduction

Colorectal cancer (CRC) is one of the most common digestive malignancies and the leading cause of cancer death in the world. In China, the incidence of CRC still continues to increase. Despite improvement in the treatment of CRC in the past decade, the overall survival of patients with CRC has not changed obviously. Metastasis is the main cause of mortalities and poor outcome [1,2]. The underlying molecular mechanisms in CRC metastasis are still unclear. Hence, it is urgent to explore key molecules in tumor progression, which may be used to design new diagnostic strategies and specific targeted drugs.

MicroRNAs (miRNAs) are a class of diverse, small, noncoding RNAs that are processed from precursors with a characteristic hairpin secondary structure [3]. They commonly function as critical gene regulators. In recent years, a large number of studies have confirmed that miRNAs are involved in tumorigenesis and metastasis by targeting various types of mRNAs [4]. To date, dysregulated expression of several miRNAs, such as miR-21 [5], miR-124 [6], miR-625 [7], miR-339-5p [8] and miR-27b [9], has been demonstrated to contribute to development and progression of CRC. In our recent study, miR-133a was identified as a tumor-suppressive factor in human CRC that acts by repressing tumor metastasis-associated protein LIM and SH3 protein 1 (LASP1) [10], which provides additional evidence of a pivotal role for miRNAs in CRC progression [11].

It has been demonstrated that that miR-1 were dysregulated and repress tumor progression in hepatocellular [12], prostate [13,14], thyroid [15], bladder [16] and renal [17]cancer. In colorectal cancer, an experimental approach, called miRNA serial analysis of gene expression (miRAGE), was used to perform the largest experimental analysis of human miRNAs. The data showed that miR-1 was down-regulation in CRC tissues with up to 11.8-fold decrease, compared with control samples [18]. Meanwhile, genome-wide profiling of chromatin signatures reveals epigenetic regulation of microRNA genes, and showed that miR-1 was methylated frequently in early and advanced colorectal cancer in which it may act as a tumor suppressor [19]. A recent study *in vitro* identified that concomitant downregulation of miR-1 and increase of metastasis-associated in colon cancer 1 (MACC1) can contribute to MET overexpression and to the metastatic behavior of colon cancer cells [20]. However, the *in vivo* function and underlying mechanism of miR-1 in CRC still have not been clarified clearly.

In this study, we detected miR-1 expression in CRC cells and tissue samples. Gain- or loss-of-function assays were performed to analyze the effect of miR-1 on tumor cell phenotypes. We established xenograft mice models to investigate its therapeutic role *in vivo*. Finally, we also explored the molecular mechanisms underlying the suppressive function of miR-1 and its potential targets.

## Materials and methods

### Cell culture and miRNA transfection

CRC cell lines HT29, HCT116, SW480, and SW620 were purchased from the American Type Culture Collection (ATCC; Manassas, Va) and maintained as previously described [10]. Additionally, a human CRC cell subline with unique liver metastatic potential, designated SW480/M5, was established in our laboratory [21] and used in the analysis. The cells were cultured in RPMI 1640 (Hyclone; Logan, Utah, USA) supplemented with 10% fetal bovine serum (FBS) (Gibco-BRL, Invitrogen; Paisley, UK) at a humidity of 5% CO_2_ at 37°C.

miRNAs were transfected at a working concentration of 100 nmol/L using Lipofectamine 2000 reagent (Invitrogen; Carlsbad, Calif, USA). The miR-1 mimic, a nonspecific miR control, anti-miR-1 (miR-1 inhibitor), and a nonspecific anti-miR control were all purchased from GenePharma (Shanghai, China). Protein and RNA samples were extracted from subconfluent cells during the exponential phase of growth.

### Tumor tissue sample

Fresh primary CRC specimens and paired noncancerous colorectal tissue were provided by the Tumor Tissue Bank of Nanfang Hospital. In each case, a diagnosis of primary CRC had been made, and the patient had undergone elective surgery for CRC in Nanfang Hospital between 2007 and 2010. The pathological diagnosis was made in the Department of Pathology of Nanfang Hospital of Southern Medical University. The study was approved by the Ethics Committee of Southern Medical University and all aspects of the study comply with the Declaration of Helsinki.

### RNA isolation, reverse transcription, and quantitative real-time PCR

See Additional file 1 (available online only) for details.

### Western blot analysis

Protein expression was assessed by immunoblot analysis of cell lysates (20–60 μg) in RIPA buffer in the presence of mouse antibodies to LIM and SH3 protein 1 (LASP1) (1:2000; Chemicon, Temecula, CA); E-cadherin, fibronectin (FN), β-actin (1:500; Santa Cruz, California, USA); rabbit antibodies to p-Akt (Ser473), p-Akt (Thr308), AKT, p44/42 MAPK (ERK1/2), p-p44/42 MAPK (ERK1/2), Rho GDP-dissociation inhibitor 1 (ARHGDIA) (1:1000; CST, Danvers, MA) and transgelin (TAGLN) (1:500; Abcam, Cambridge, UK).

### Cell proliferation assays

See Additional file 1 (available online only) for details.

### Cell migration analysis

See Additional file 1 (available online only) for details.

### Preparation of lentiviral vectors

A DNA fragment corresponding to pre–miR-1 and the flanking sequence was amplified from human genomic DNA and then cloned into pGLV3/H1/GFP + puro lentiviral vector (http://www.genepharma.com). The production, purification, and titration of lentivirus were performed as described by *Liu* and colleagues [22]. The packaged lentiviruses were named LV-miR-1. The empty lentiviral vector LV-con was used as a control.

### Tumor growth assay

See Additional file 1 (available online only) for details.

### Tumor metastasis assays

See Additional file 1 (available online only) for details.

### Proteomic analysis

See Additional file 1 (available online only) for details.

### Bioinformatics

Potential miRNA targets were predicted and analyzed using 3 publicly available algorithms: PicTar, TargetScan, and miRanda [23]. The number of false-positive results was decreased by accepting only putative target genes that were predicted by at least 2 programs.

### miRNA target validation

A 2992-bp fragment of the *LASP1* 3’ untranslated region (3’UTR) was amplified by PCR and cloned downstream of the firefly luciferase gene in the psiCHECK-2 vector (Promega; Madison, Wis, USA). This vector was named wild-type (wt) 3’UTR. Site-directed mutagenesis of the miR-1 binding site in the *LASP1* 3’UTR was carried out using the GeneTailor Site-Directed Mutagenesis System (Invitrogen) and named mutant (mt) 3’UTR. For reporter assays, the wt or mt 3’UTR vector and miR-1 mimic or inhibitor were cotransfected. Luciferase activity was measured 48 h after transfection using the Dual-Luciferase Reporter Assay System (Promega, Madison, Wis, USA).

### Statistical analysis

Data were analyzed using SPSS version 13.0 software (SPSS; Chicago, Ill, USA). The Student *t*-test and the one-way ANOVA test were carried out for qRT-PCR and CCK-8 analyses and to calculate the tumor growth curve. The correlation between miR-1 and LASP1 was determined using the Spearman rank correlation test. Statistical significance was established at *P* < 0.05.

## Results

### Decreased expression of miR-1 in CRC tissues and cell lines

**Figure 1:**
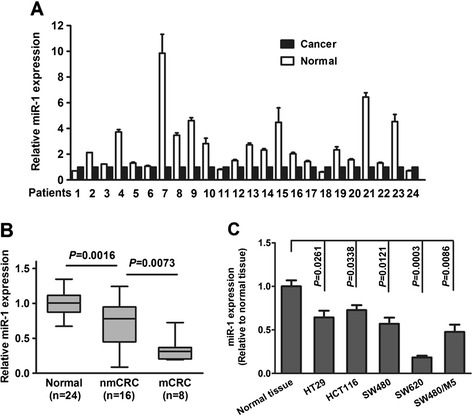
**The expression of miR-1 was decreased in CRC tissues and cell lines. (A)** The histogram indicates the differential expression of miR-1 in cancerous versus non-cancerous tissue using qRT-PCR. **(B)** The miR-1 expression in CRC tissues with or without metastases relative to match-normal tissues. nmCRC denotes CRC tissues without metastases; mCRC denotes CRC tissues with metastases. **(C)** The relative expression of miR-1 in five CRC cell lines (SW480, SW620, HCT116, SW480/M5, and HT29) was significantly decreased compared with the mean rate of expression of miR-1 in 24 non-cancerous tissue samples (N-tissue).

Real-time PCR were used to detect miR-1 expression in 24 CRC tissue and matched adjacent non-cancerous tissue. Down-regulation of miR-1 expression was found in 20 of all the CRC samples, with up to 9.8-fold decrease, compared with control samples (Figure 1A). The expression of miR-1 were significantly decreased in CRC tissues than control samples (*P* = 0.0016; Figure 1B). A relatively lower level was found in metastatic CRC (mCRC) compared with non-metastatic CRC (nmCRC) (*P* = 0.0073; Figure 1B). In addition, lower expression of miR-1 was found in all five CRC cell lines compared with the mean level of the non-cancerous tissue specimens. In consistent with the data of Figure 1B, a higher expression of miR-1 was found in SW620 and SW480/M5 cells with higher metastasis potential compared with SW480, HCT116 and HT29 cells derived from the primary tumors (Figure 1C).

### Exogenous miR-1 suppressed CRC cell proliferation and migration *in vitro*

**Figure 2:**
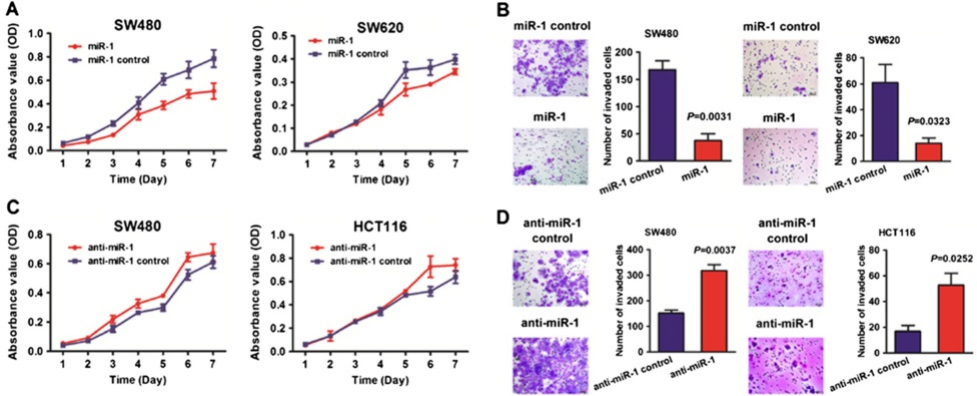
**Ectopic expression of miR-1 inhibited aggressive phenotypes in CRC cells. (A)** The effect of miR-1 on cell proliferation was evaluated by CCK-8 assay after miR-1 transfection of SW480 and SW620 cells. **(B)** Data of transwell assay for SW480 and SW620 cells. The cells were counted under a microscope in five randomly selected fields. Bars represent the number of cells invaded after transfection with miR-1. **(C)** The effect of anti-miR-1 on cell proliferation was evaluated by CCK-8 assay after anti-miR-1 transfection of SW480 and HCT116 cells. **(D)** Data of transwell assay for SW480 and HCT116 cells. The cells were counted under a microscope in five randomly selected fields. Bars represent the number of cells invaded after transfection with anti-miR-1.

We transfected the CRC cell lines SW480 and SW620 with miR-1 mimic and evaluated the effects on cellular behaviors. Real-time PCR were performed to detect the transfection efficiency (*P* < 0.05; Additional file 2: Figure S1). CCK8 assays revealed a significant reduction in the proliferative ability of miR-1-transfected SW480 and SW620 cells, respectively (*P* < 0.05; Figure 2A). Transwell assays showed that miR-1 significantly decreased the potential of cell migration in SW480 and SW620 (*P* < 0.05; Figure 2B).

On the contrary, anti-miR-1, as a miRNA inhibitor, was used to investigate the role of miR-1 depletion in CRC cell lines SW480 and HCT116. On the contrary with the above results, we found a significant increase in the ability of cell proliferation in SW480 and HCT116, respectively (P < 0.05; Figure 2C). Introduction of anti-miR also enhanced the number of invaded cells after transfection with miR-1 inhibitor (*P* < 0.05; Figure 2D). These findings suggest miR-1 as a suppressor inhibits aggressive phenotype of CRC cells.

### Endogenous overexpression of miR-1 inhibited CRC growth and metastasis *in vivo*

**Figure 3:**
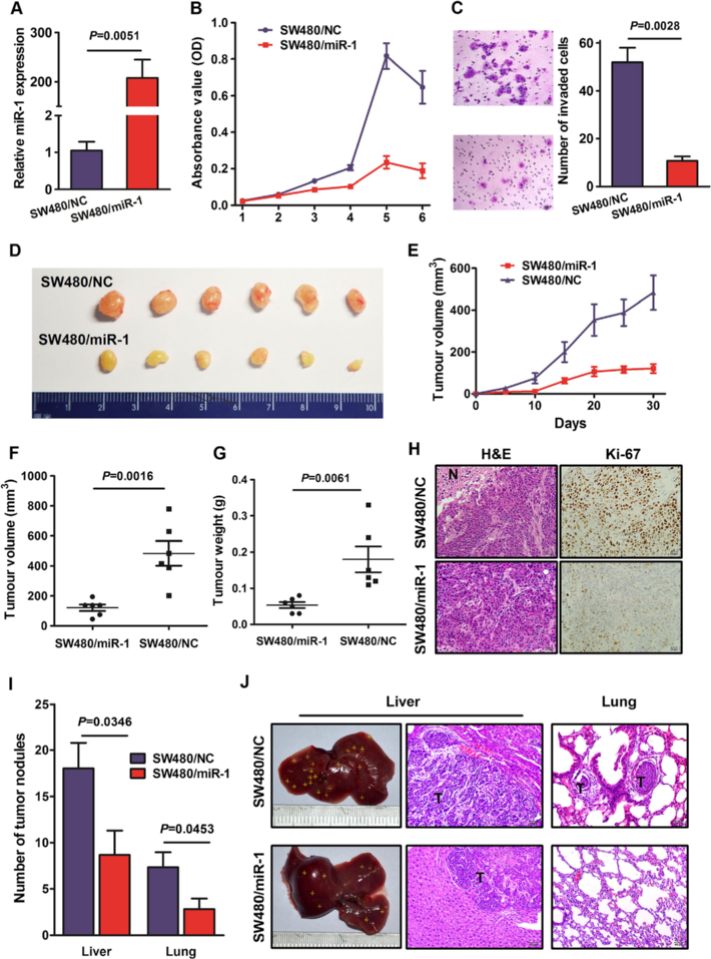
**miR-1 inhibited tumor growth and metastasis in nude mice. (A)** The histogram indicates the increased expression of miR-1 in SW480 cells with miR-1 overexpression using qRT-PCR. **(B)** Cell proliferation was evaluated by CCK-8 assay between SW480/miR-1, which has stable overexpression of miR-1 and control SW480/miR-NC cells. **(C)** Representative figures and data of transwell assay for SW480/miR-1 and SW480/NC cells. Each bar represents the mean ± SD. The results were reproduced in three independent experiments. **(D)** Tumor cells were injected subcutaneously into the back of nude mice to evaluate cancerogenesis. Representative figure of tumors formed. **(E)** Tumor volume in the back of nude mice injected with SW480/miR-1 and SW480/NC cells was measured. The data of all primary tumors are expressed as mean ± SD. **(F)** Scatter plots of tumor volume derived from SW480/miR-1 and SW480/NC cells at 30 d after subcutaneous implantation. **(G)** Scatter plots of tumor weight derived from SW480/miR-1 and SW480/NC cells at 30 d after subcutaneous implantation. **(H)** Representative photographs of haematoxylin and eosin **(H & E)** and immunohistochemical staining of primary cancer tissues were shown (×400). N, necrotic lesion. **(I)** Number of liver and lung nodules per mice. The number of tumor nodules in individual mice was counted under the microscope. **(J)** Tumour cells were injected into nude mice through the spleen and tail vein to evaluate the lung and liver homing capacity of cells, respectively (**H & E** staining, ×200). The yellow crosses indicated tumor nodules in the liver and lung. T, tumor lesions.

We infected SW480 cells with LV-miR-1 and then established SW480/miR-1 cell line with stable miR-1 overexpression. A qRT-PCR assay confirmed up-regulated 208-fold of miR-1 expression in SW480/miR-1 cells compared with SW480/miR-NC cells (Figure 3A). *In vitro* assay showed that stable overexpression of miR-1 obviously decreased the potential of cell growth and migration (Figure 3B-C). Then, a subcutaneous tumor model was used to evaluate the effect of miR-1 on tumorigenesis of CRC cells. As shown in Figure 3D, the tumors in the SW480/miR-1 group grew more slowly than these in the SW480/miR-NC group, and showed significantly lower Ki-67 index compared with control (Figure 3E-H).

To analyze the relationship between the potential of homing capacity and miR-1 expression, we observed liver and lung nodules after injection of tumor cells via spleen and tail vein, respectively. Compared with SW480/miR-1 group, we found significantly more and larger tumor nodules in the liver and lung of SW480/miR-NC group, indicating that miR-1 inhibited the homing capacity of CRC cells (Figure 3I-J).

### miR-1 changed protein expression pattern of CRC cells

**Figure 4:**
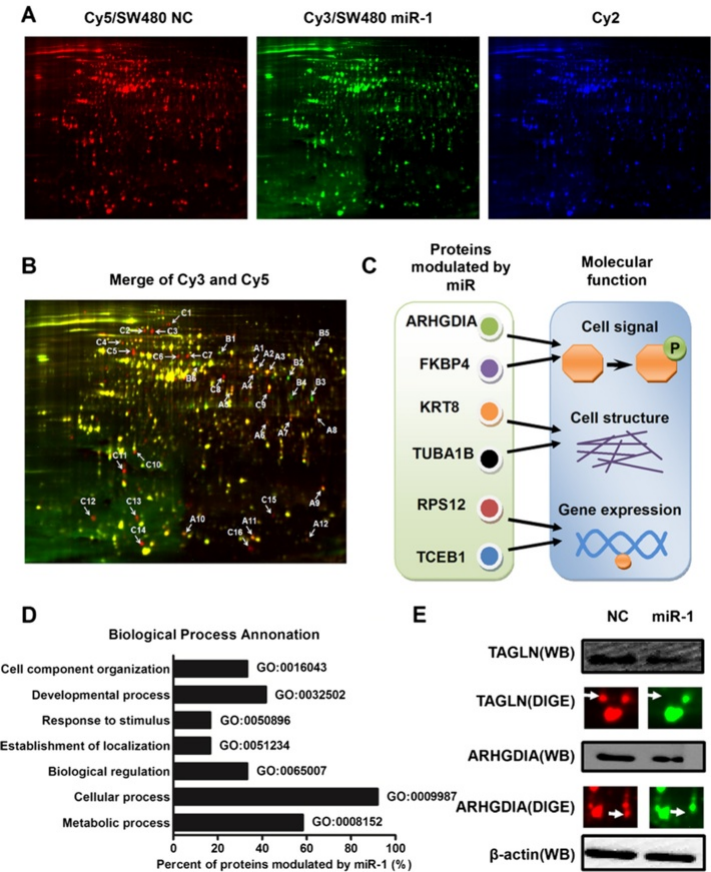
**miR-1 altered global protein expression profiles and involved in several key biological processes. (A)** 2-D DIGE images of SW480 cells transfected with miR-1 are shown. Proteins from cells transfected with control were labelled with Cy5. Proteins from cells transfected with miR-1 were labelled with Cy3. Internal standard proteins were labelled with Cy2. **(B)** Distribution of differentially expressed protein spots in merged images of the Cy-dye labelled images is shown. **(C)** Diagrammatic sketch showed major molecular functions exerted by miR-1 using Gene Ontology. **(D)** The histogram indicated main biological processes involved in miR-1 using Gene Ontology. **(E)** Both enlarged DIGE images and immunoblotting results of two candidate protein, identified as ARHGDIA and TAGLN, are shown.

To reveal the underlying molecular mechanisms of biological behaviors mediated by miR-1, we performed two dimensional differential gel electrophoresis (2D-DIGE) based proteomics strategy to exhibit differential expression protein profiling after transfection with miR-1 in SW480 cells (Figure 4A). Using software analysis, 33 differential protein spots were found. Among of them, total 31 protein spots were successfully identified by matrix-assisted laser desorption/ ionization tandem time of flight mass spectrometry (MALDI-TOF/TOF MS) (Figure 4B; Additional file 3: Table S1). Two candidate proteins, identified as Rho GDP-dissociation inhibitor 1 (ARHGDIA) and transgelin (TAGLN), was confirmed by western blot analysis, suggesting that the results of proteomic analysis are convincing (Figure 4E).

We next explore the biological processes involved in proteins modulated by miR-1 using Gene Ontology. All of the proteins were integrated into several key biological processes, such as development, response to stimulus, localization and metabolism, *et al.* (Figure 4C). Molecular function annotation indicated that miR-1 may participate in cell signal transduction, regulation of gene expression and cytoskeleton reorganization, *et al.* (Figure 4D). This suggests that miR-1 may play an important role in tumor progression through diverse mechanisms.

### miR-1 mediated inhibition of epithelial-mesenchymal transition (EMT) and inactivation of signal transduction pathway

**Figure 5:**
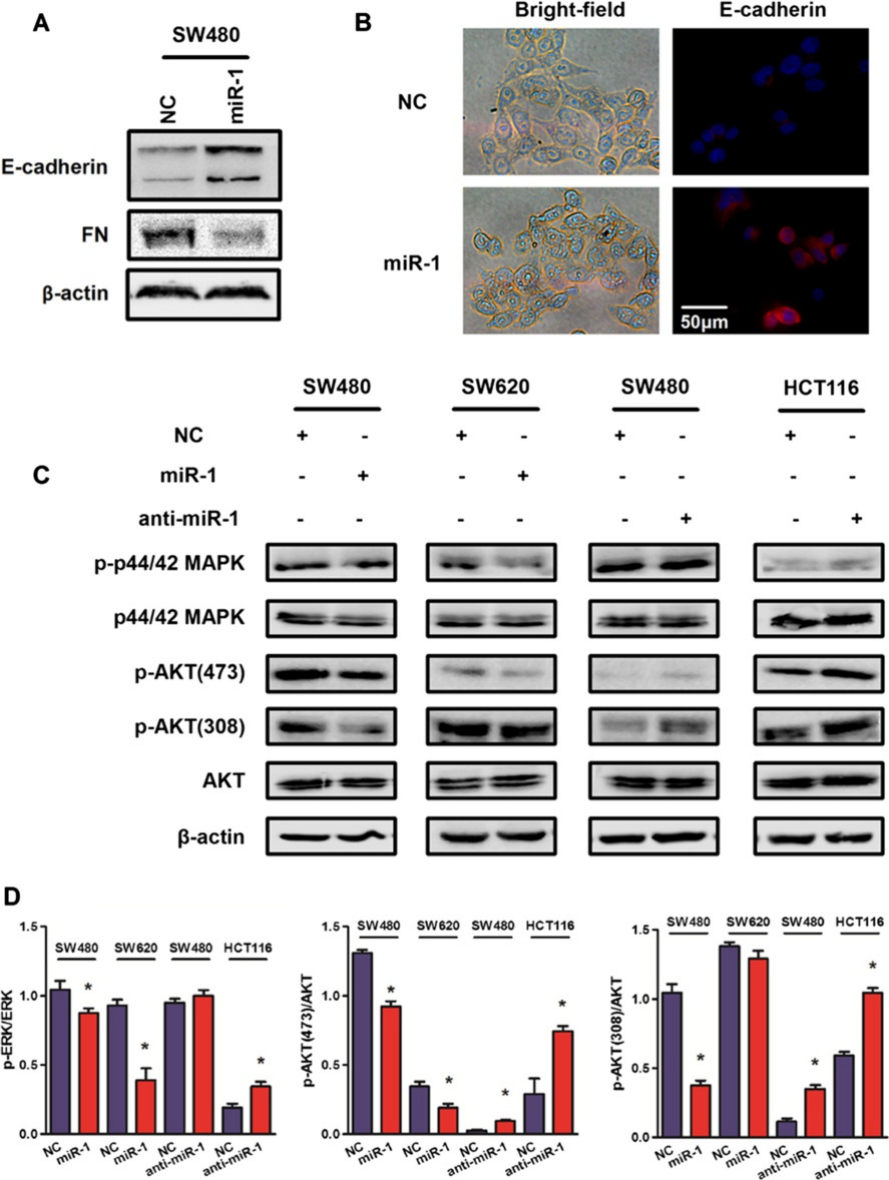
**miR-1 inhibited EMT and suppressed phosphorylation of ERK and AKT. (A)** Western blot analysis was performed to detect the expression of epithelial cell marker E-cadherin and mesenchymal markers FN in SW480 cells transfected with miR-1. **(B)** The light microscopy was used to observe the change of cell morphology in SW480 cells after treatment with miR-1. The immunofluorescence (IF) assay was used to detect the expression of E-cadherin. **(C)** Phosphorylation levels of ERK and AKT were detected in SW480, HCT116 and SW620 cells transfected with miR-1 or anti-miR-1. **(D)** The immunosignal was quantified using densitometric scanning software, and relative protein abundance was determined by normalisation with total levels of ERK and AKT, respectively.

EMT is an critical process during tumor metastasis by which epithelial cells acquire mesenchymal properties and show reduced intercellular adhesion and increased motility [24]. Because we have observed miR-1-mediated reduction of migration ability and gene expression regulation, we asked whether miR-1 introduction have an impact on the expression of EMT-associated proteins. Western blot assays showed that exogenous miR-1 resulted in increase of epithelial markers E-cadherin and decrease of mesenchymal marker fibronectin (FN) (Figure 5A). Using immunofluorescence (IF) assay, we observed an epithelial-like changes and increased expression of E-cadherin in SW480 cells after treatment with miR-1 (Figure 5B).

Further, we carried out western blot analysis of the phosphorylation status of proteins involved in EMT signaling. As shown in Figure 5C, miR-1 significantly inhibited mitogen-activated protein kinases (MAPK) pathway through dephosphorylation of p44/42 MAPK (ERK1/2) and phosphatidylinositol 3-kinase (PI3K)/AKT signaling via decreased phosphorylation of AKT at Ser473 and Thr308 in SW480 and SW620 cells. On the contrary, anti-miR-1 activated MAPK and PI3K/AKT pathways via phosphorylation of ERK1/2 and AKT.

### miR-1 directly targets tumor metastasis related gene LASP1 in CRC cells

**Figure 6:**
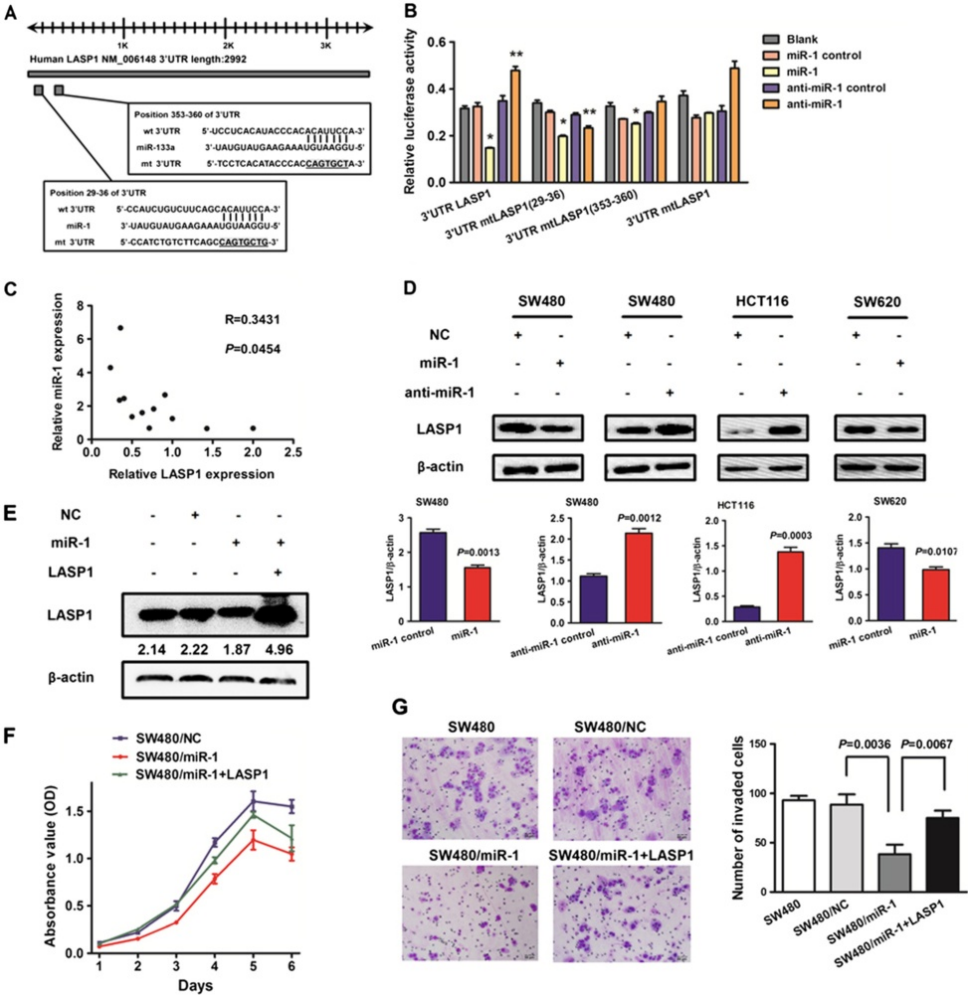
**LASP1 was a direct target of miR-1 in CRC cells. (A)** Diagram of LASP1 3’UTR containing 2 putative conserved target sites for miR-1, which were identified using the TargetScan database. **(B)** Results of luciferase reporter assays in 293 T cells, with cotransfection of wt or mt 3’UTR and miR mimic and inhibitor, as indicated. **(C)** Expression of miR-1 and LASP1 mRNA were detected in CRC tissues by qRT-PCR analysis. A statistically significant inverse correlation between miR-1 and LASP1 mRNA was observed in CRC specimens. **(D)** LASP1 protein expression in SW480, SW620, and HCT116 cells 48 hours after transfection with miR-1 or anti-miR-1 was detected by Western blot analysis. The immunosignal was quantified using densitometric scanning software, and relative protein abundance was determined by normalization with β-actin. Each bar represents the mean ± SD. The results were reproduced in 3 independent experiments. The asterisk (*) indicates P < 0.05. **(E)** SW480 cells transfected with miR-1 mimic and/or LASP1 cDNA were used to determine the role of LASP1 in miR-1–mediated biological behaviors. Expression of LASP1 protein was detected by Western blot analysis. **(F)** Cell proliferation was determined by CCK-8 assay. **(G)** Results of transwell assay, which was carried out to evaluate the effect of cell migration after transfection are shown.

Using a bioinformatic analysis (based on TargetScan Human 6.2, PicTar and miRanda), LIM and SH3 protein 1 (LASP1), identified previously as a CRC metastasis related gene, was predicted as a possible target of miR-1. Luciferase reporter assay was performed to determine whether miR-1 can directly target the 3’UTR region of Lasp1. This involved in cloning the target sequence (wt 3’UTR) or mutant sequence (mt 3’UTR) into a luciferase reporter vector then transfecting 293 T cells with the wt or mt 3’UTR vector and miR-1 mimic or inhibitor (Figure 6A). A significant decrease of luciferase activity was detected in wt vectors after transfected with miR-1 mimic. A mutation in the putative binding site in the Lasp1 3’UTR region abrogated this repression, thereby providing additional evidence of direct interaction between miR-1 and 3’UTR region of Lasp1 (Figure 6B). mRNA expression by both miR-1 and LASP1 was analysed by qRT-PCR in CRC tissue samples. LASP1 mRNA levels in CRC tissues had a negative correlation with miR-1 (Figure 6C). The result was confirmed further during our investigation of miR-1 for its ability to suppress LASP1 mRNA and protein expression in CRC cells (Figure 6D).

To determine whether LASP1 rescue the suppressive effect of miR-1, we simultaneously co-transfected SW480 cells with miR-1 mimic and pcDNA3-LASP1 vector (which contained all but the 3’UTR of LASP1) (Figure 6E). We found that the ectopic expression of LASP1 partly neutralized down-regulation of miR-1 on LASP1 expression and rescued miR-1-mediated inhibition of cell proliferation and migration (Figure 6F-G).

## Discussion

The expression of miRNAs was abnomal in various kinds of human cancer [25,26]. More and more researches has documented that miRNAs play essential roles in multiple biological processes, including cell differentiation, proliferation, angiogenesis, invasion and migration [27-30]. Recently, several studies have showed the deregulation of miR-1 in many types of tumor, such as hepatocellular [12], prostate [13,14], thyroid [15], bladder [16] and renal [17] cancer. In colorectal cancer, genome-wide profiling of chromatin signatures reveals epigenetic regulation of microRNA genes, and showed that miR-1 was methylated frequently in early and advanced colorectal cancer in which it may act as a tumor suppressor [19]. Our data demonstrated that down-regulation of miR-1 was frequently exist in CRC tissue and cell lines, suggesting a tumor suppressive role of miR-1 in CRC development.

Until now, no functional evidence *in vivo* of miR-1 has been documented in CRC. In prostate cancer, miR-1 inhibited cell proliferation, migration and invasion by suppressing the expression of purine nucleoside phosphorylase (PNP) [14]. A follow-up study showed that miR-1 was reduced in patients with distant metastasis and recurrence, and may further serve as an independent prognostic factor [13]. A recent study *in vitro* identified that concomitant downregulation of miR-1 and increase of metastasis-associated in colon cancer 1 (MACC1) can contribute to MET overexpression and to the metastatic behavior of colon cancer cells [20]. Similarly, our gain- and loss-of-function assay showed that miR-1 suppressed cell proliferation and migration *in vitro*. Importantly, our findings showed that miR-1 reduced tumor growth and metastasis *in vivo*, suggesting its suppressive role in CRC progression.

The molecular mechanisms underlying miR-1-mediated biological behaviors are still unclear. To comprehensively understand the effect of miR-1 on cancer cells, we performed proteomic analysis to screen the alteration of protein profiling in CRC cells. Using 2-D DIGE, we found a set of proteins that might be directly or indirectly modulated by miR-1. The candidate proteins have been reported involved in tumor development and progression. Interestingly, our previous study has demonstrated that a candidate protein, ARHGDIA, was upregulated in metastatic CRC and promoted cell migration of CRC cells [31]. Another candidate protein, identified as TAGLN, has been demonstrated as a potential target of miR-1 using TargetScan software. Similarly, TAGLN2 has been reported as a target of miR-1 in renal cell carcinoma [17], bladder cancer [16] and head and neck squamous cell carcinoma [32]. These findings are consistent with our proteomic results and a suppressive role of miR-1 in CRC.

Epithelial–mesenchymal transition (EMT) plays a pivotal role in the initiation of metastasis, a process in which epithelial cells lose adhesion and cytoskeletal components concomitant with a gain of mesenchymal components and the initiation of a migratory phenotype [33,34]. Because the effect of miR-1 on cell migration and gene expression regulation, we detected the change of EMT markers in SW480 cells transfected with miR-1. The results supported that miR-1 may reverse EMT process to inhibit cell migration. Similarly, miRNA-1 suppresses prostate cancer metastasis by regulating epithelial-mesenchymal transition [35]. Further study showed that Slug is a major regulator of mesenchymal differentiation and directly represses miR-1 transcription. Slug and miR-1/-200 act in a self-reinforcing regulatory loop, leading to amplification of EMT.

Our proteomic analysis revealed that miR-1 might be associated with cell signaling pathway. The findings of immunoblot assays demonstrated that miR-1 inactivated MAPK/ERK and PI3K/AKT pathway by dephosphorylation of ERK1/2 and AKT, which is classical signal transduction pathway and plays an essential role in tumor progression. Recent researches revealed that the activation of cap-dependent translation by cooperative ERK and AKT signaling is critical for promotion of CRC motility and metastasis. Inhibition of either ERK or AKT alone showed limited activity in inhibiting cell migration and invasion, but combined inhibition resulted in significant impact [36]. To the best of its knowledge, we firstly demonstrated miR-1 exerted its biological functions by regulation of MAPK/ERK and PI3K/AKT pathway, which may also explain miR-1-mediated reversion of EMT process.

miRNAs generally exert their biological function by suppressing their specific target genes at a posttranscriptional level. A number of mRNAs were reported as direct targets of miR-1, such as transgelin 2 (TAGLN2) [16,32], purine nucleoside phosphorylase (PNP) [14,32], coding for the cyclin D2 (CCND2), CXC chemokine receptor 4 (CXCR4), stromal cell derived factor-1 (SDF-1) [15], endothelin-1 [12] and fibronectin1 [37] *et al*. In the research, we validated the targeting of LASP1 (a tumor metastasis-associated protein in CRC), showing that miR-1 may suppress tumors by binding directly in the LASP1 3’UTR. LASP1 is a specific focal adhesion protein involved in numerous biological and pathological processes [38,39]. Overexpression of LASP1 has been described in several types of cancers [40-42]. In a previous study, we demonstrated overexpression of LASP1 in metastatic CRC tissue and found that the expression of this protein correlated closely with the overall survival of patients with CRC. RNA interference-mediated silencing of LASP1 in SW620 CRC cells inhibited cell proliferation and migration significantly. However, gene transfection-mediated overexpression of LASP1 in SW480 CRC cells resulted in aggressive cancer cell phenotypes and promoted cancer growth and metastasis (in contrast with the phenotypes induced by miR-1 restoration). These results show that LASP1 might be a promising target in developing treatments for patients with CRC [10]. LASP1 overexpression can rescue miR-1-mediated biological activity. These results suggest that the inhibitory effect of miR-1 on the biological activity and MAPK or AKT signal pathway is mediated in part through the repression of LASP1 expression.

Taken together, the identification of miR-1 as a tumor-suppressive miRNA in human CRC that acts by repressing LASP1 provides x evidence of a pivotal role for miRNAs in CRC tumorigenesis and progression. Given that miR-1 is down-regulated in CRC, the re-introduction of this mature miRNA into tumor tissue could serve as a therapeutic strategy by reducing the expression of target genes. miRNA-based therapeutics are still in their infancy; however, our findings are encouraging and suggest that this miRNA could be targeted for the development of a treatment for patients with CRC, especially metastatic CRC, in the future.

## Additional files

Additional file 1: **Supplementary Materials and Methods.** RNA isolation, reverse transcription, and quantitative real-time PCR.

Additional file 2: Figure S1. The histogram indicates the increased expression of miR-1 in SW480 **(A)** and SW620 **(B)** cells transfected with miR-1 using qRT-PCR.

Additional file 3: Table S1. The differential proteins indentified by MS.

## Abbreviations

miRNA: MicroRNA
LASP1: LIM and SH3 protein 1
CRC: Colorectal carcinoma
EMT: Epithelial-mesenchymal transition
FN: Fibronectin
ARHGDIA: Rho GDP-dissociation inhibitor 1
TAGLN: Transgelin
FBS: Fetal bovine serum
IHC: Immunohistochemistry
NC: Negative control
miRNA: MicroRNA
PCR: Polymerase chain reaction
PI3K: Phosphatidylinositol-4,5-bisphosphate 3-kinase
UTR: Untranslated region
MAPK: Mitogen-activated protein kinase
MEK: MAP kinase kinase
AKT: Protein kinase B
ERK: Extracellular signal–regulated kinase
