# Supplementary material for: Tumor suppressor miR-1 restrains epithelial-mesenchymal transition and metastasis of colorectal carcinoma via the MAPK and PI3K/AKT pathway
Source: J Transl Med. 2014 Sep 8;12:244. doi: 10.1186/s12967-014-0244-8 (PMC4172896; doi:10.1186/s12967-014-0244-8)
Supplement: Supplementary file 1 — Supplementary Materials and Methods. RNA isolation, reverse transcription, and quantitative real-time PCR. [file 12967_2014_244_MOESM1_ESM.docx]

**Supplementary Materials and Methods**

**RNA isolation, reverse transcription, and quantitative real-time PCR**

Total RNA was extracted using Trizol reagent (Invitrogen). To quantitate miR-1 expression, total RNA was polyadenylated and underwent reverse transcription using an NCode miRNA First-Strand cDNA Synthesis kit (Invitrogen). mRNA levels in the Lasp1 gene were measured as previously described ([Zhao et al 2010](#_ENREF_1)). qRT-PCR was carried out using an SYBR Green PCR master mix (Applied Biosystems; Foster City, Calif, USA) on an ABI 7500HT system. *GAPDH* or *U6* snRNA was used as an endogenous control. All samples were normalized to internal controls, and fold changes were calculated through relative quantification (2^-ΔΔCT^).

**Cell proliferation assays**

Cell proliferation assays were carried out using Cell Counting Kit 8 (CCK8) (Dojindo; Kumamoto, Japan). Cells were plated in 96-well plates at a density of 1 × 10^4^ cells per well and cultured in the growth medium. At the indicated time points, the number of cell in triplicate wells was measured using the absorbance at 450 nm of reduced WST-8 (2-(2-methoxy-4-nitrophenyl)-3-(4-nitrophenyl)-5-(2,4-disulfophenyl)-2H-tetrazolium,monosodium salt).

**Cell migration analysis**

Cells from the serum-free medium (1 × 10^5^ cells/100 μL) were added to the top chamber of each 8-mm–pore transwell chamber (Corning Star; Cambridge, Mass, USA). The bottom chamber was prepared using 10% FBS as a chemoattractant. Cells were allowed to migrate through the porous membrane for 20 h at 37℃. The cells that stuck to the lower surface of the membrane were treated with a ﬁxation/staining solution (0.1% crystal violet, 1% formalin, and 20% ethanol) for visualization. The cells were counted under a microscope in 5 randomly selected ﬁelds (original magniﬁcation, ×200). At least 4 chambers from 3 different experiments were analyzed.

**Tumor growth assay**

Female BALB/c nude mice aged 4 to 5 weeks were purchased from the Laboratory Animal Services Centre of the Southern Medical University. Animal handling and experimental procedures were approved by the Animal Experimental Ethics Committee of Southern Medical University. For the tumor growth assay, 1 × 10^6^ SW480/miR-1 cells and SW480/miR-NC cells were independently injected subcutaneously into the left back of nude mice (n = 6/group). After the injection, the ﬂuorescence emitted by the cells was imaged using a whole-body GFP imaging system (Lighttools; Encinitas, Calif, USA). IPP5.0 software (Cybermetics; Silver Spring, Md, USA) was used to analyze whole-body optical images that showed real-time tumor growth and tumor volume. The tumor volume was calculated using the following formula: V = 0.5 × D × d^2^, where V represents volume, D represents the longitudinal diameter, and d represents the latitudinal diameter.

**Tumor metastasis assays**

To determine the lung and liver metastatic potential of cancer cells in vivo, we injected 5 × 10^6^ SW480/miR-1 cells and SW480/miR-NC cells into nude mice (n = 5/group) through the tail vein and spleen, respectively. Whole-body optical images were obtained to monitor primary tumor growth and the formation of metastatic lesions. The mice were all sacrificed 2 months later, at which time individual organs were removed and metastatic tissue was analyzed using hematoxylin and eosin (H & E) and immunohistochemical (IHC) staining.

**Proteomic analysis**

Two-dimensional fluorescence difference gel electrophoresis (2D DIGE) and mass spectrometry (MS) were carried out as previously described ([Zhao et al 2010](#_ENREF_1)). The proteins were labeled with ﬂuorescent cyanine dyes (GE Healthcare, Milwaukee, Wis, USA) following the manufacturer’s instructions. Electrophoresis was carried out in the dark, and the tests for biological function were run on 3 gels. An additional strip was prepared in parallel to pick spots as described in 2D DIGE, except that the isoelectric point (IPG) strip was loaded with 1000 mg of proteins and the gel was stained with Coomassie brilliant blue. After SDS-PAGE, the 3 gels were scanned using a Typhoon 9410 scanner (GE Healthcare; Madison, Wis, USA) with appropriate excitation/emission wavelengths speciﬁc for Cy2 (488/520 nm), Cy3 (532/580 nm) and Cy5 (633/670 nm) to generate 9 protein spot maps.

DeCyder 5.0 software (GE Healthcare) was used for 2D DIGE analysis according to the manufacturer’s recommendation. The DeCyder differential in-gel analysis (DIA) module was used to make a pairwise comparison of each sample with the internal standard in each gel. The DeCyder biological variation analysis (BVA) module was then used to match all 9 protein spot maps simultaneously using the Cy3:Cy2 and Cy5:Cy2 DIA ratio to calculate the mean change in abundance and the paired Student *t* test to find *P* values for the variance of these ratios for each protein pair across all samples. The protein spots (ratio >2, *P* < .05) that were altered consistently in all 9 protein spot maps were selected for further identiﬁcation.

**References**

Zhao L, Wang H, Liu C, Liu Y, Wang X, Wang S *et al* (2010). Promotion of colorectal cancer growth and metastasis by the LIM and SH3 domain protein 1. *Gut* **59:** 1226-1235.
