# Supplementary material for: Tumor suppressor miR-1 restrains epithelial-mesenchymal transition and metastasis of colorectal carcinoma via the MAPK and PI3K/AKT pathway
Source: J Transl Med. 2014 Sep 8;12:244. doi: 10.1186/s12967-014-0244-8 (PMC4172896; doi:10.1186/s12967-014-0244-8)
Supplement: Supplementary file 2 — The histogram indicates the increased expression of miR-1 in SW480 (A) and SW620 (B) cells transfected with miR-1 using qRT-PCR. [file 12967_2014_244_MOESM2_ESM.docx]

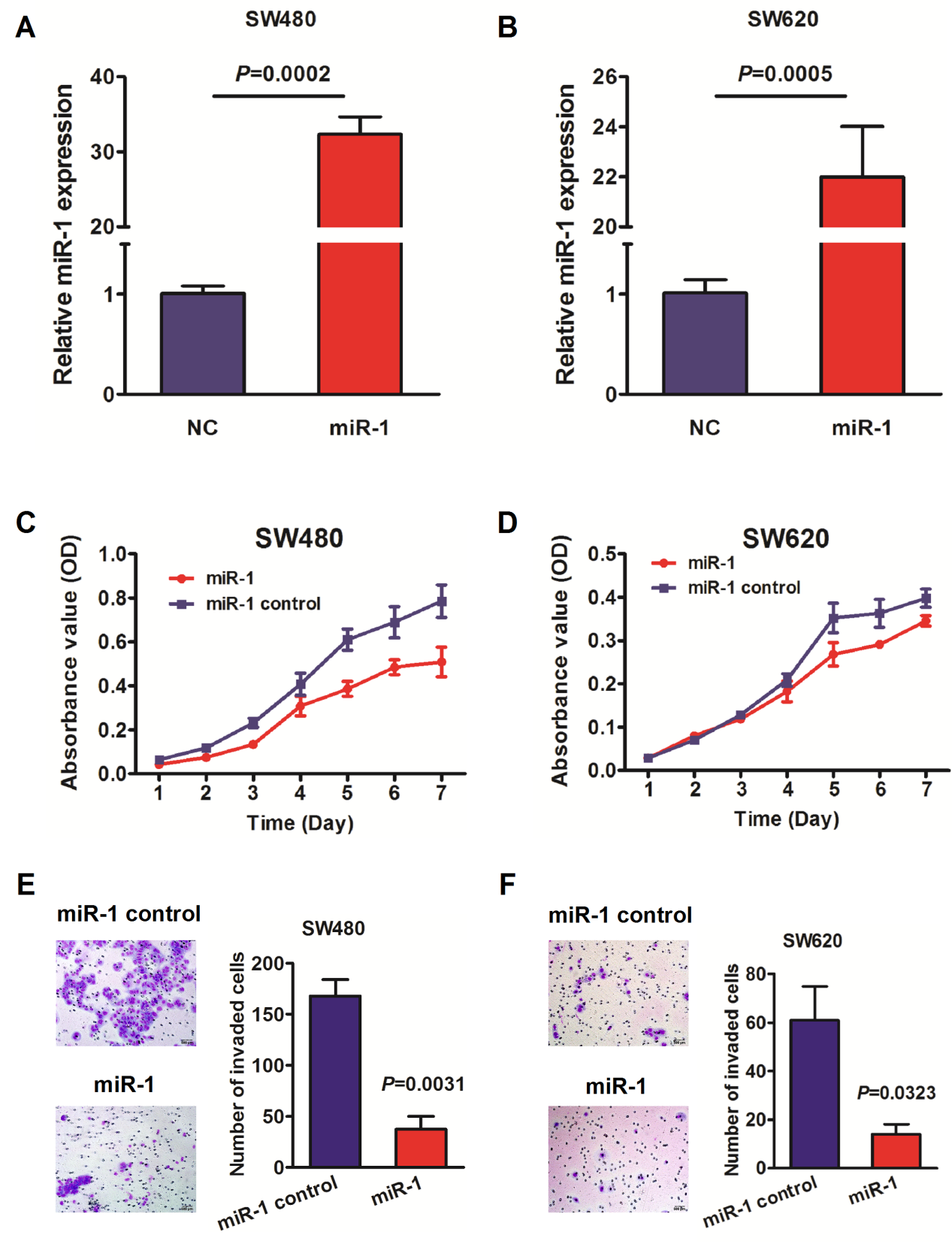


Supplementary Figure 1. The histogram indicates the increased expression of miR-1 in SW480 (A) and SW620 (B) cells transfected with miR-1 using qRT-PCR.
