# Supplementary material for: Tumor suppressor miR-1 restrains epithelial-mesenchymal transition and metastasis of colorectal carcinoma via the MAPK and PI3K/AKT pathway
Source: J Transl Med. 2014 Sep 8;12:244. doi: 10.1186/s12967-014-0244-8 (PMC4172896; doi:10.1186/s12967-014-0244-8)
Supplement: Supplementary file 3 — The differential proteins indentified by MS. [file 12967_2014_244_MOESM3_ESM.doc]

**Supplementary Table 1.** The differential proteins indentified by MS

| **No.^a^** | **Accession no.** | **Protein name** | **Abbreviations** | **Theoretical** | | **Mascot socre** | **Coverage rate** | **Gene Ontology (GO)** | **Protein level** |
| --- | --- | --- | --- | --- | --- | --- | --- | --- | --- |
|  |  |  |  | **Mass** | **p*I*** |  |  |  |  |
| A1 | CENPE_HUMAN | Isoform 3 of Centromere-associated protein E | CENPE | 302971.1 | 5.27 | 82.6 | 29.88% | microtubule motor activity | Down |
| A2 | NDE1_HUMAN | Isoform 2 of Nuclear distribution protein nudE homolog 1 | NDE1 | 37868.84 | 4.79 | 59.5 | 44.48% | protein binding | Down |
| A3 | DYST_HUMAN | DST Uncharacterized protein | DST | 865400.28 | 4.89 | 73.5 | 15.89% | actin binding | Down |
| A4 | TADBP_HUMAN | Isoform 1 of TAR DNA-binding protein 43 | TARDBP | 45053.44 | 6.07 | 333 | 41.79% | nucleotide binding | Down |
| A5 | K22E_HUMAN | Keratin, type II cytoskeletal 2 epidermal | KRT2 | 66110.5 | 8.17 | 130 | 52.56% | structural constituent of cytoskeleton | Down |
| A6 | CCD57_HUMAN | Isoform 2 of Coiled-coil domain-containing protein 57 | CCDC57 | 85584.62 | 5.50 | 90.8 | 38.62% | protein binding | Down |
| A7 | Unidentified |  |  |  |  |  |  |  | Down |
| A8 | PSA3_HUMAN | proteasome subunit alpha type-1 isoform 3 | PSMA3 | 14728.57 | 8.76 | 370 | 73.85% | protein binding | Down |
| A9 | CHD8_HUMAN | CHD8 Protein | CHD8 | 102633.67 | 8.29 | 63.7 | 25.32% | p53 binding | Down |
| A10 | K2C1_HUMAN | Keratin, type II cytoskeletal 1 | KRT1 | 66170.07 | 8.33 | 81.2 | 36.80% | structural constituent of cytoskeleton | Down |
| A11 | CX6B1_HUMAN | Cytochrome c oxidase subunit 6B1 | COX6B1 | 12255.7 | 7.38 | 134 | 54.37% | cytochrome-c oxidase activity | Down |
| A12 | PCDP1_HUMAN | Primary ciliary dyskinesia protein 1 | PCDP1 | 64609.41 | 9.09 | 74.3 | 39.82% | calmodulin binding | Down |
| B1 | PDIA3_HUMAN | Protein disulfide-isomerase A3 | PDIA3 | 57145.9 | 6.28 | 453 | 69.31% | protein binding | Up |
| B2 | K1C10_HUMAN | Keratin, type I cytoskeletal 10 | KRT10 | 59019.78 | 4.88 | 73.4 | 42.29% | structural molecule activity | Up |
| B3 | MELT_HUMAN | Isoform 2 of Ventricular zone-expressed PH domain-containing protein homolog 1 | VEPH1 | 90425.6 | 6.71 | 76.3 | 34.52% | phospholipid binding | Up |
| B4 | DYST_HUMAN | Isoform 2 of Dystonin | DST | 593805.68 | 5.40 | 73.6 | 17.13% | actin binding | Up |
| B5 | STIP1_HUMAN | STIP1 protein | STIP1 | 68721.43 | 7.83 | 226 | 40.85% | protein binding | Up |
| B6 | K2C8_HUMAN | Keratin, type II cytoskeletal 8 | KRT8 | 53671.13 | 5.26 | 600 | 62.73% | structural molecule activity | Up |
| C1 | Unidentified |  |  |  |  |  |  |  | Absence |
| C2 | CENPE_HUMAN | Isoform 1 of Centromere-associated protein E | CENPE | 317587.86 | 5.36 | 83.6 | 31.62% | microtubule motor activity | Absence |
| C3 | MYH15_HUMAN | Myosin-15 | MYH15 | 225904.23 | 5.62 | 87.6 | 31.71% | actin binding | Absence |
| C4 | K2C1_HUMAN | Keratin, type II cytoskeletal 1 | KRT1 | 66170.07 | 8.33 | 66.3 | 21.27% | structural constituent of cytoskeleton | Absence |
| C5 | TBA1B_HUMAN | Tubulin alpha-1B chain | TUBA1B | 50803.86 | 4.70 | 270 | 47.01% | structural constituent of cytoskeleton | Absence |
| C6 | FKBP4_HUMAN | Peptidyl-prolyl cis-trans isomerase FKBP4 | FKBP4 | 52057.18 | 5.11 | 469 | 66.23% | protein binding | Absence |
| C7 | FKBP4_HUMAN | Peptidyl-prolyl cis-trans isomerase FKBP4 | FKBP4 | 52057.18 | 5.11 | 556 | 77.12% | protein binding | Absence |
| C8 | MAP6_HUMAN | cDNA FLJ41346 fis, clone BRAWH2005315 | MAP6 | 51297.01 | 9.62 | 71.8 | 39.88% | negative regulation of microtubule depolymerization | Absence |
| C9 | SYNE1_HUMAN | Isoform 7 of Nesprin-1 | SYNE1 | 646262.26 | 5.65 | 106 | 21.67% | actin binding | Absence |
| C10 | GDIR1_HUMAN | Rho GDP-dissociation inhibitor 1 | ARHGDIA | 23249.73 | 4.74 | 408 | 44.61% | cellular component movement | Absence |
| C11 | PLPL9_HUMAN | Phospholipase A2, group VI | PLA2G6 | 21942.65 | 8.09 | 53.1 | 55.84% | phospholipase A2 activity | Absence |
| C12 | ELOC_HUMAN | TCEB1 Uncharacterized protein | TCEB1 | 10067.76 | 5.10 | 294 | 85.39% | protein binding | Absence |
| C13 | COX5A_HUMAN | Cytochrome c oxidase subunit 5A, mitochondrial | COX5A | 16922.75 | 6.78 | 114 | 52.67% | electron carrier activity | Absence |
| C14 | TAGL_HUMAN | Transgelin | TAGLN | 12312.45 | 6.95 | 76 | 84.33% | actin binding | Absence |
| C15 | RS12_HUMAN | 40S ribosomal protein S12 | RPS12 | 14904.63 | 7.27 | 201 | 70.45% | structural constituent of ribosome | Absence |

a Spot numbers refer to those in Figure 5B

b Change of normalized spot intensity between SW480 cells transfected with miR-1 mimic versus miR-1 NC
